# Improving the performance of ghost imaging via measurement-driven framework

**DOI:** 10.1038/s41598-021-86275-2

**Published:** 2021-03-24

**Authors:** Hanqiu Kang, Yijun Wang, Ling Zhang, Duan Huang

**Affiliations:** 1 School of Automation, Central South University, Changsha, 410083 China; 2 School of Computer Science and Engineering, Central South University, Changsha, 410083 China

**Keywords:** Imaging and sensing, Displays

## Abstract

High-quality reconstruction under a low sampling rate is very important for ghost imaging. How to obtain perfect imaging results from the low sampling rate has become a research hotspot in ghost imaging. In this paper, inspired by matrix optimization in compressed sensing, an optimization scheme of speckle patterns via measurement-driven framework is introduced to improve the reconstruction quality of ghost imaging. According to this framework, the sampling matrix and sparse basis are optimized alternately using the sparse coefficient matrix obtained from the low-dimension pseudo-measurement process and the corresponding solution is obtained analytically, respectively. The optimized sampling matrix is then dealt with non-negative constraint and binary quantization. Compared to the developed optimization schemes of speckle patterns, simulation results show that the proposed scheme can achieve better reconstruction quality with the low sampling rate in terms of peak signal-to-noise ratio (PSNR) and mean structural similarity index (MSSIM). In particular, the lowest sampling rate we use to achieve a good performance is about 6.5%. At this sampling rate, the MSSIM and PSNR of the proposed scheme can reach 0.787 and 17.078 dB, respectively.

## Introduction

As a novel and non-local imaging technique, ghost imaging (GI) was first experimentally demonstrated by using quantum entanglement source generated by the spontaneous parameter down-conversion (SPDC) process^1^. Considering the difficulty of generating quantum entanglement source, it was proved in theory and experiment that GI could be implemented with thermal and pseudo-thermal light^2–8^. In recent years, with the proposal of various GI schemes^6,9–11^, GI has a bright application prospect in many related fields, such as remote sensing^12,13^, imaging through scattering media^14–17^, optical encryption^18,19^ and terahertz imaging^20–24^.

In a typical GI system, the target image can be obtained by correlation measurement between two distributed light beams^1,2^. Due to the existence of background noise, the signal-to-noise ratio (SNR) and visibility of the reconstructed image are lower than expected^25,26^. To address these issues, various schemes have been proposed and verified, among which differential GI (DGI)^27^ and normalized GI (NGI)^28^ demonstrate significant improvement in the SNR though at the cost of more measurements; intensity threshold^29^ and time–space-average domain^30^ schemes show that the visibility of the image restoration can be remarkable enhanced. Compressed sensing (CS)^31^ theory combined with GI technique^9,32,33^ can not only improve the performance of the image reconstruction, but also reduce the number of measurements. In addition, the sampling efficiency can be further improved, which is helpful to promote the practical application of GI. In general, the GI methods based on CS usually work better than traditional GI based on the second-order correlation when the sparsity is considered in the process of image restoration^34^. Deep learning-based GI schemes^10,11,17,35^ have also been proved to be effective in improving sampling efficiency and imaging quality. Furthermore, the imaging speed is faster compared with conventional GI method.

Despite that many schemes have been proposed to improve performance of GI from different perspectives, the problem of sampling efficiency still cannot be ignored^36,37^. The high sampling efficiency means that enough information about the target can be obtained with a few samplings, and then high-quality reconstruction can be achieved. Therefore, how to improve the sampling efficiency deserves further study in GI. In fact, the problem of enhancing sampling efficiency can be transformed into the speckle pattern optimization problem, that is, the sampling matrix optimization problem, which can learn from the matrix design methods in CS^36–40^. So far, various speckle patterns have been designed to optimize the light fields of GI, such as multi-scale speckle patterns^36^, sinusoidal patterns^37^ and hadamard patterns^41^. Furthermore, mutual coherence minimization^39^ and dictionary learning^40^ schemes have also been proved to be reasonably effective. It should be pointed out that the dictionary learning scheme can be regarded as using a data-driven framework or a task-driven framework^42^. Under this framework, the sparse coefficient matrix is obtained from high-dimensional training data and updated along with the sparse basis. Different from the dictionary learning scheme that optimizes the speckle patterns through the learned sparse basis or dictionary from a given training set^40^, in this paper we propose a novel optimization scheme from the perspective of optimizing the sampling matrix and sparse basis, which is based on the measurement-driven framework (MDF)^43^. Under the MDF, the sparse coefficient matrix is obtained by low-dimensional pseudo-measurement process and updated separately from the sparse basis.

In this work, we formulate the optimization problem of sampling matrix and sparse basis in GI as an Frobenius-norm minimization problem, and then propose an alternating optimization algorithm to solve the design problem and obtain the corresponding analytical solution. The proposed scheme also suggests a new binary quantization threshold for the optimized sampling matrix, which can effectively overcome the influence of quantization error. Compared with grayscale speckle patterns, binary speckle patterns refresh faster on the digital micro-mirror device (DMD)^44^, which may be used in some aspects of GI. We perform a simulation using given dataset to test the feasibility of proposed method, and evaluate the performance under different sampling rates and noise levels. Results show that our proposal can achieve the high imaging quality at low sampling rate.

## Methods

### Problem formulation

Mathematically, the sampling process in GI can be expressed as^45^

1 $$\begin{aligned} {{y}} = {\Phi }{{x}}+{\varepsilon }\end{aligned}$$

where ${{y}}$ is an *M* dimension column vector that denotes the signal measured by the bucket detector in the signal arm, ${\Phi }$ is an $M \times N$ sampling matrix detected by the detector in the reference arm and preserves the light field intensity information, ${{x}}$ is an *N* dimension column vector that stands for the object to be reconstructed, ${\varepsilon }$ represents the additive noise.

According to the CS theory, the object ${{x}}$ can be approximately described in the form of

2 $$\begin{aligned} {{x}} = {\Psi }{{z}}+{{e}} \end{aligned}$$

where ${\Psi }$ is a certain overcomplete sparse basis, ${{z}}$ is called sparse coefficient vector, ${{e}}$ is referred to as the sparse representation error (SRE). Denote ${{X}}$ and ${{Z}}$ as the training dataset and sparse coefficient matrix, respectively, as

3 $$\begin{aligned} {{X}}(:,p) = {{x}}_{p}, \quad {{Z}}(:,p) = {{z}}_{p}, \quad p = 1, \cdots , P \end{aligned}$$

here, *P* represents the number of training samples. And then, the total SRE is given by

4 $$\begin{aligned} {{E}} = {{X}} - {\Psi }{{Z}} \end{aligned}$$

The recent works in CS have shown that the accuracy of image reconstruction can be improved in designing a sensing matrix with consideration of possible SRE^43,46,47^. Motivated by these works, we first introduce the SRE into the optimization of speckle patterns in GI with the MDF. Next, we present our scheme in detail.

Let sampling matrix ${\Phi }$ be constrained by the form^43^

5 $$\begin{aligned} {\Phi }={{U}}\left[ {{I}}_{M}\,\,{{0}}\right] {{V}}^{\top } \end{aligned}$$

where ${{I}}_{M}$ is an *M*-dimensional unit matrix, ${{U}}$ and ${{V}}$ are two arbitrary orthonormal matrices of dimensions $M \times M$ and $N \times N$, respectively. A matrix restricted by Eq. (5) is actually a tight frame with good expected-case performance for CS applications^48^. Then, additional non-negative constraints are imposed on elements of the sampling matrix to meet the requirement of light field intensity, namely,

6 $$\begin{aligned} {\Phi }_{m,n} \ge 0 \end{aligned}$$

Inspired from the problem formulation in^43^, we combine the SRE with the above sampling matrix constraint conditions in our scheme. The following objective function to be minimized for optimizing sampling matrix and sparse basis is proposed:

7 $$\begin{aligned} \begin{array}{cl} \min _{{\Phi }, {\Psi }} &{} \rho ({\Phi }, {\Psi }) = \Vert ({{I}}_{N}-\omega {\Phi }^{\top } {\Phi })({{X}}-{\Psi } {{Z}})\Vert _{F}^{2} \\ \text{ subject } \text{ to } &{} {\Phi }= {{U}}\left[ {{I}}_{M}\,\,{{0}}\right] {{V}}^{\top } \text{ and } \text{ then } {\Phi }_{m, n} \ge 0 \end{array} \end{aligned}$$

where ${{I}}_{N}$ is an *N*-dimensional unit matrix, $\Vert \cdot \Vert _{F}$ is the Frobenius-norm, $\omega$ is a trade-off factor with the value of (0,1).

### Optimization of sampling matrix and sparse basis based on MDF

We propose an alternating optimization algorithm via MDF to solve the optimization problem in Eq. (7). It consists of two processes, namely the pseudo-measurement process and the alternating optimization process. Note that we define the measurement process of the training samples as the pseudo-measurement process, in order to distinguish it from the measurement process of the target images or test set in our scheme. In fact, given the training samples, sampling matrix and sparse basis, we first acquire a pseudo measure of the training samples through randomly initialized sampling matrix. Then, according to the pseudo-measurement results, sampling matrix and sparse basis, a certain reconstruction algorithm is used to obtain the sparse coefficient matrix, for example using orthogonal matching pursuit (OMP)^51^. However, the actual measurement process is to sense the specific targets with the optimized sampling matrix. After obtaining the sparse coefficient matrix, sampling matrix and sparse basis are then updated alternately. The above two processes are specifically as follows.

Given the training dataset ${{X}}$, sampling matrix ${\Phi }_{0}$, sparse basis ${\Psi }_{0}$, sparsity level *K*, number of iterations *Iter* and trade-off factor $\omega$. Firstly, pseudo-measurement process. With the pseudo-measurement result ${{Y}} = {\Phi }_{0} {{X}}$, solve the following problem:

8 $$\begin{aligned} \widehat{{{Z}}}=\arg \min _{{{Z}}} \; \Vert {{Y}}-{\Phi }_{0} {\Psi }_{0} {{Z}}\Vert _{F}^{2} \quad \text{ subject } \text{ to } \quad \Vert {{Z}}(:, p)\Vert _{0} \le K, p = 1, \cdots , P \end{aligned}$$

where $\Vert \cdot \Vert _{0}$ is the $\ell _{0}$-norm. Then sparse coefficient matrix $\widehat{{{Z}}}$ is obtained by OMP algorithm^51^. Secondly, alternating optimization process. For *i* from 1 to *Iter*, with ${\Phi }_{i-1}$ fixed, update sparse basis ${\Psi }_{i}$ by:

9 $$\begin{aligned} {\Psi }_{i}=\arg \min _{{\Psi }_{i}} \; \rho ({\Phi }_{i-1}, {\Psi }_{i}) \end{aligned}$$

then, with ${\Psi }_{i}$ fixed, update sampling matrix ${\Phi }_{i}$ by:

10 $$\begin{aligned} {\Phi }_{i}=\arg \min _{{\Phi }_{i}} \; \rho ({\Phi }_{i}, {\Psi }_{i}) \quad \text{ subject } \text{ to } \quad {\Phi }_{i} = {{U}}\left[ {{I}}_{M}\,\,{{0}}\right] {{V}}^{\top } \end{aligned}$$

Obviously, with OMP algorithm, the $\widehat{{{Z}}}$ can be obtained by the low-dimension pseudo-measurement ${{Y}}$, rather than the high-dimension training dataset ${{X}}$, which is different from data-driven or task-driven frameworks adopted in dictionary learning problems^42^. The detailed updating processes of sparse basis and sampling matrix are shown in Supplementary S1 and S2, respectively.

### Constraints on the optimized sampling matrix

After getting the optimized sampling matrix ${\widehat{{\Phi }}}$, we need to perform the following two steps on the sampling matrix:

- Non-negative constraint. Considering the non-negativity of speckle patterns displayed on the DMD, We need to impose the following constraint on the sampling matrix:
  11 $$\begin{aligned} \widehat{{\Phi }}_{m,n} = 0 \quad \text{ if } \widehat{{\Phi }}_{m,n} < 0. \end{aligned}$$
- Binarization threshold optimization. Compared with grayscale patterns, binary patterns have significant advantages in data storage and transmission. Moreover, binary patterns refresh faster on the DMD, which is extremely important in real-time ghost imaging applications^44^. Different from the previous binary threshold selection strategies^49,50^, an effective binarization threshold is provided to quantize the sampling matrix. Let $\alpha > 0$ be the binarization threshold, which is defined as the average of all non-zero elements of the optimized sampling matrix, then
  12 $$\begin{aligned} \widehat{{\Phi }}_{m,n}=\left\{ \begin{array}{ll} 1, &{} \text{ if } \widehat{{\Phi }}_{m,n} \ge \alpha , \\ 0, &{} \text{ if } \widehat{{\Phi }}_{m,n} < \alpha . \end{array}\right. \end{aligned}$$

We notice that the (1, − 1) patterns are also selected to generate measurement results^17^. Compared with the (1, −1) patterns, the optimized non-negative speckle patterns can significantly reduce memory requirement. Moreover, it can be loaded directly onto the DMD while the (1, −1) patterns are in an indirect way. More importantly, it may be used to generate the (1, −1) patterns and produce better recovery quality in comparison with the (1, −1) patterns.

## Results

### Imaging schematic diagram

**Figure 1:**
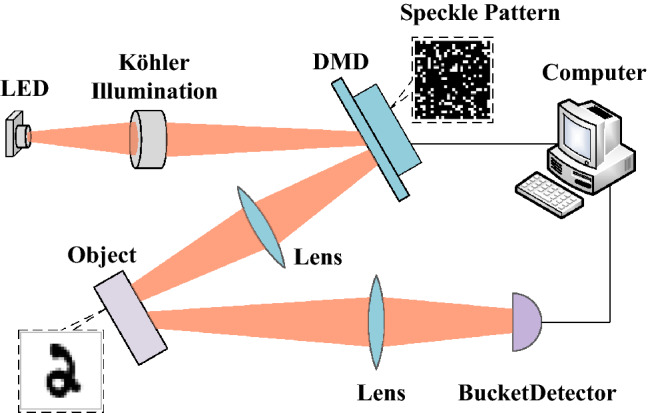
Conceptual diagram of a computational ghost imaging system. The light is spatially shaped via a DMD into speckle patterns which illuminate the object. The overall scattered light from the sample is acquired via a bucket detector and the signal is feed into the optimisation loop.

The schematic diagram of ghost imaging system is shown in Fig. 1. Through the Köhler illumination system, the light generated by a light-emitting diode (LED) is evenly projected on the DMD. After being modulated by the optimized speckle patterns preloaded on the DMD, the reflected light from the DMD is projected on the object to be measured through a lens. Then, the reflected light from the specific target is collected by the lens and eventually recorded by the bucket detector.

### Imaging reconstruction

The validity of the proposed scheme via MDF is verified by simulations. All the simulations are performed in MATLAB R2016b. In our simulations, the MNIST database^53^ is selected to generate the training data, where pixel values of each digit image with a size of $28 \times 28$ are normalized to the range of 0 to 1. We first randomly select 5000 digital images from the training set to optimize the sampling matrix and sparse basis. Next, the optimized sampling matrix is constrained by Eq. (11) and Eq. (12). Then, each row of the sampling matrix is reshaped into a speckle pattern consistent with the size of imaging object. Finally, the speckle pattern on the DMD is approximately regarded as the light field intensity distribution on the object plane. In each sampling process the result of dot product between the speckle pattern and imaging object is obtained and then all the intensity values of the result are summed as the measurement result of the bucket detector. We compare the proposed method with the unoptimized case and Xu’s method^39^. For convenience, we denote the optimized ghost imaging scheme based on the measurement-driven framework as OGIMDF, and the corresponding unoptimized case without MDF is denoted as UGI. The characteristics of these methods are summarized in Table 1.

**Table 1:** The characteristics of three schemes in terms of sampling matrix and its constraints and sparse basis.

Method	Sampling matrix	Sparse basis	Constraints of ${\Phi }_{m,n}$
Xu^39^	Matrix optimization	DCT	Eq. (11) and Eq. (12)
UGI	Eq. (5)	Random Gaussian	Eq. (11) and Eq. (12)
OGIMDF	Matrix optimization	Matrix optimization	Eq. (11) and Eq. (12)

For Xu’s method^39^, the Discrete Cosine Transform (DCT) basis is used as the sparse representation basis, while random Gaussian matrices with a Gaussian distribution of zero-mean and unit variance are chosen as sparse basis for UGI method. The sampling matrices of all test methods are subjected to non-negative constraint in Eq. (11) and binary threshold quantization in Eq. (12). For UGI and OGIMDF methods, the images are reconstructed by solving the following problem:

13 $$\begin{aligned} \widehat{{{X}}}=\arg \min _{{{X}}^{\prime }} \frac{1}{2}\Vert {{Y}}^{\prime }-\widehat{{\Phi }} {{X}}^{\prime }\Vert _{F}^{2}+\lambda \Vert {{X}}^{\prime }\Vert _{1} \quad \text{ subject } \text{ to } \quad {{Y}}^{\prime } = \widehat{{\Phi }} {{X}}_{test} \end{aligned}$$

via the fast iterative shrinkage-threshold algorithm (FISTA)^52^, where $\lambda > 0$ is a regularization parameter, $\Vert \cdot \Vert _{1}$ is the $\ell _{1}$-norm, ${{X}}_{test}$ is chosen from MNIST test set, and each column of it can be reshaped into a target image. The reconstruction algorithm for Xu’s scheme is selected according to the corresponding reference.

### Simulation results

**Figure 2:**
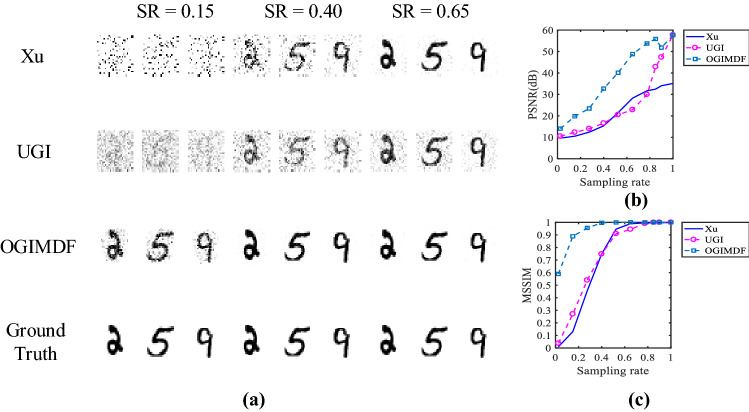
Simulation results of three schemes under different SRs. (**a**) The images in the first row are reconstructed using Xu method, the second row shows the images reconstructed using UGI, the third row shows the restored objects using OGIMDF, and the images in the last row are the ground truth. (**b**) and (**c**) show the change curves of PSNR and MSSIM of reconstructed images using Xu, UGI and OGIMDF under different SRs, respectively.

Figure 2 presents the simulation results with various schemes. The corresponding time cost of matrix optimization and reconstruction is recorded in Table 2. The reconstruction time of each method is the average of the total reconstruction time of 500 test images under the corresponding sampling rate. The reconstruction images at the given sampling rate are shown in the Fig. 2a. The sampling rate (SR) is defined as the ratio of the number of samplings to the number of image pixels. Figure 2b,c show the peak signal-to-noise ratio (PSNR) and mean structural similarity (MSSIM) index curves of the restored images with respect to SR, respectively. As the quantitative metrics of the reconstructed image quality, PSNR and MSSIM^54^ are defined as follows:

14 $$\begin{aligned} {\text {PSNR}}(F,G)=\,& {} 10 \log _{10}\left( \frac{H^{2}}{{\text {MSE}}(F,G)}\right) \end{aligned}$$

15 $$\begin{aligned} {\text {MSE}}(F,G)=\,& {} \frac{1}{N} \sum _{j}^{N}\left( F_{j}-G_{j}\right) ^{2} \end{aligned}$$

16 $$\begin{aligned} {\text {MSSIM}}(F,G)=\,& {} \frac{1}{W} \sum _{j}^{W} {\text {SSIM}}(f_{j},g_{j}) \end{aligned}$$

where *F* and *G* are the original image and reconstructed image, respectively, *H* is the maximum value of the image pixels and $H = 255$ in this paper, *N* is the total number of the image pixels, *W* is the number of local windows in the image, $f_{j}$ and $g_{j}$ are the corresponding windows with a size of $14 \times 14$ pixels. The structural similarity (SSIM) is given by

17 $$\begin{aligned} {\text {SSIM}}\left( f_{j}, g_{j}\right) =\frac{\left( 2 \mu _{fj} \mu _{gj}+c_{1}\right) \left( 2 \sigma _{fgj}+c_{2}\right) }{\left( \mu _{fj}^{2}+\mu _{gj}^{2}+c_{1}\right) \left( \sigma _{fj}^{2}+\sigma _{gj}^{2}+c_{2}\right) } \end{aligned}$$

where $(\mu _{fj}, \sigma _{fj})$ and $(\mu _{gj}, \sigma _{gj})$ are the means and variances of $f_{j}$ and $g_{j}$, respectively, $\sigma _{fgj}$ is the co-variance of $f_{j}$ and $g_{j}$, $c_{1} = (0.01 H)^{2}$ and $c_{2} = (0.03H)^{2}$. We calculate the averaged values of PSNR and MSSIM of 500 reconstructed images and then plot the corresponding curves.

**Table 2:** Running time of three methods in terms of matrix optimization and image reconstruction.

Method	Matrix optimization (s)	Reconstruction (s)
Xu^39^	0.654 to 4.310(for 100 iterations)	0.478 to 1.284
UGI	–	1.137 to 1.828
OGIMDF	37.347 to 43.722(for 5 iterations)	1.210 to 1.802

As seen from the Fig. 2, the recovered performance of the proposed OGIMDF is overall better than the other schemes. Compared with the UGI method, the proposed scheme can significantly improve the PSNR and MSSIM, especially at the low SR, mainly owing to the optimization of the sampling matrix. We can also see that the MSSIM for the Xu’s method performs comparably with the UGI scheme under different SRs, but the performance of PSNR in the Xu’s method is inferior to the UGI scheme when the SR exceeds 0.775. The main reason is the relatively large reconstruction error for the Xu’s method at the high SR ranges. It can also be seen from Fig. 2a that the reconstructed image quality of the proposed scheme is clearer than that of the other two schemes at the given SR.

**Figure 3:**
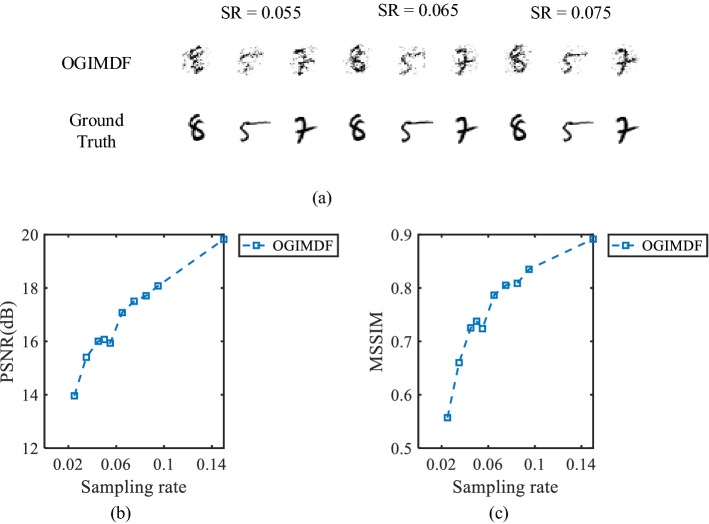
Simulation results of the OGIMDF method at the low SRs. The SR is between 0.025 and 0.15. (**a**) The images in the first row are reconstructed using OGIMDF method, and the images in the second row are the ground truth. (**b**) and (**c**) show the change curves of PSNR and MSSIM of reconstructed images using OGIMDF under different SRs, respectively.

We study the performance of the proposed scheme at low SRs, which is what we are concerned about in practice. We present the performance of the OGIMDF scheme when the SR is between 0.025 and 0.15, and the results are shown in Fig. 3. In the Fig. 3a, the reconstructed images at the given SR are displayed. Fig. 3b,c show the averaged performance of PSNR and MSSIM of 500 restored images with regard to SR, respectively. As shown in Fig. 3, when the SR is less than 0.065, the image quality of the OGIMDF method is poor and the recovered images are blurred and damaged. With the increase of SR, the imaging quality is improved and the recovered images can be recognized. Therefore, we think that 0.065 may be the lowest SR that the OGIMDF method can achieve in the case of good imaging quality.

**Figure 4:**
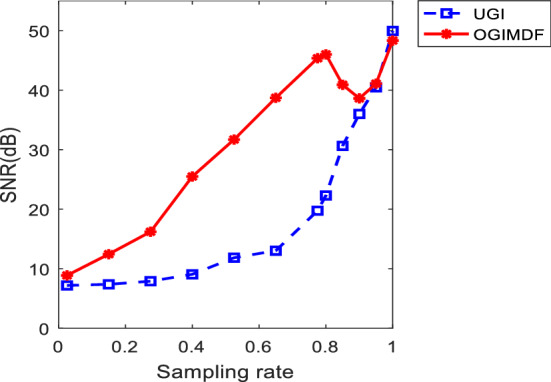
The SNR curves of reconstructed images with respect to SR for UGI and OGIMDF schemes.

We note that the PSNR of the proposed scheme decreases slightly when the SR is between 0.80 and 0.90 in Fig. 2b. To explain this phenomenon, we draw the SNR curves of the reconstructed images for OGIMDF and UGI schemes at the different SRs, as shown in Fig. 4. For the OGIMDF scheme, the SNR has a similar downward trend with the PSNR in Fig. 2b when the SR ranges from 0.80 to 0.90. This means that the noise of the reconstructed images is enhanced in this SR region. However, the PSNR and SNR values of the proposed scheme are still higher than the UGI scheme in this SR range, and begin to increase again when the SR is more than 0.90, both of which prove the superiority of our proposal.

**Figure 5:**
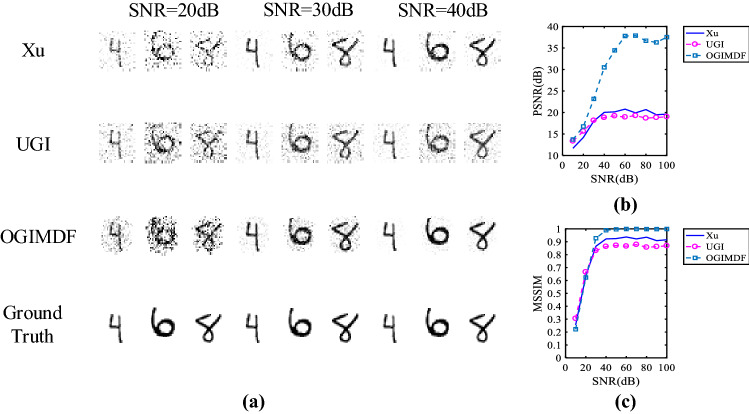
Simulation results of three schemes under different SNRs with SR = 0.50. (**a**) The images in the first row are reconstructed using Xu method, the second row shows the images reconstructed using UGI, the third row shows the restored objects using OGIMDF, and the images in the last row are the ground truth. (**b**) and (**c**) show the change curves of PSNR and MSSIM of reconstructed images using Xu, UGI and OGIMDF under different SNRs, respectively.

The above simulations do not take into account the impact of additional noise on the reconstruction performance. In the practical environment, noise is inevitable. To evaluate the influence of noise on the three schemes, we add Additive White Gaussian Noise (AWGN) to the bucket values. Fig. 5 presents the corresponding results of various schemes when SR is 0.50. In the Fig. 5a, the reconstructed images at the given SNR are displayed. Figure 5b,c show the averaged performance of PSNR and MSSIM of 500 restored images with regard to SNR, respectively. From the Fig. 5b,c, we can find that when the SNR does not exceed 20 dB, the PSNR values of three methods all lie in the relatively low level and the MSSIM of the UGI behaves slight advantages among these schemes. In the Fig. 5a, as a result, the images reconstructed by UGI, OGIMDF and Xu’s methods are blurred to a certain extent when SNR is 20 dB. Obviously, under the low SNR, the useful information is destroyed by noise, so it is difficult to restore the original image clearly. However, with the growth of SNR, both the PSNR and MSSIM of the OGIMDF accelerate rapidly. When SNR exceeds 30 dB, the OGIMDF begin to establish its superiority, i.e., when SNR is 40 dB, the PSNR values of UGI, OGIMDF and Xu’s methods are 18.83 dB, 30.50 dB, 20.04 dB, respectively. Moreover, the MSSIM value of the OGIMDF is 7.30$\%$ higher than that of the Xu’s method at this SNR. Accordingly, from the Fig. 5a, we can also see that the reconstruction results of the OGIMDF are remarkably clearer than the other results. Therefore, in practical application, the noise acting on the bucket detector should be reduced as much as possible to obtain high quality reconstruction performance for the OGIMDF.

**Figure 6:**
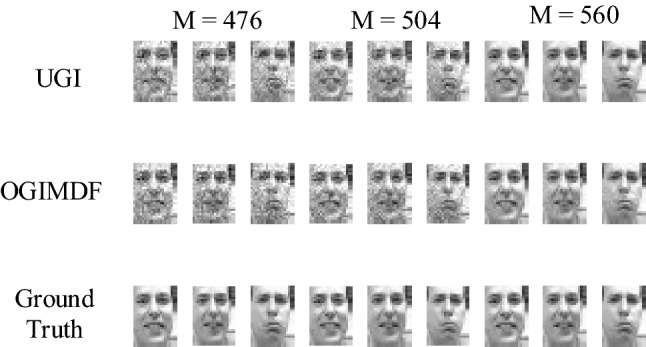
Reconstruction results of grayscale targets under different sampling numbers for UGI and OGIMDF schemes. M represents the sampling number.

We further study the reconstruction quality of grayscale targets under different sampling numbers. The Frey faces database^55^ including 1965 face images with a size of $28\times 20$ is selected to generate training and test data. In our simulation, 1915 faces randomly selected from the database are used as training samples, and the remaining faces are used for testing. Figure 6 presents the simulation results of UGI and OGIMDF schemes, and the sampling numbers are 476, 504 and 560, respectively. As shown in Fig. 6, with the increase of the number of sampling, the quality of the reconstructed image is also improved. when the sampling number is 560, the two methods can both reconstruct the image clearly. This verifies that the proposed scheme is also capable of reconstructing grayscale images.

**Figure 7:**
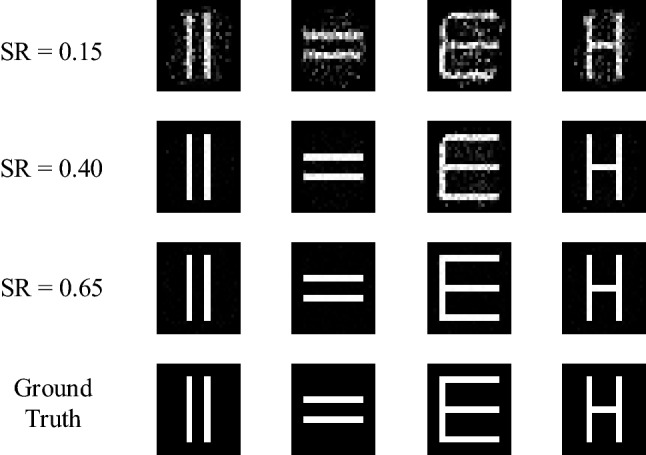
The test results of extensibility for the OGIMDF method under different SRs using the English letters and the double-seam patterns.

We also verify the extensibility of the proposed method. The training data contains 5000 images randomly selected from the MNIST database^53^. We test the proposed scheme with English letters and double-seam patterns, and give the reconstruction results in the Fig. 7. We can see that it can still reconstruct well the English letters and the double-seam patterns when the SR is 0.15. At this SR, the PSNR and MSSIM are 17.47 dB and 0.85, respectively. When the SR increases to 0.65, the reconstruction images of the English letters and the double-seam patterns become clearer. These results well demonstrate the extensibility of the proposed scheme.

## Conclusion

In this paper, we have proposed an optimization scheme of speckle patterns via measurement-driven framework to improve the reconstruction quality of GI. We have analyzed the performance of the proposed scheme under different SRs by using PSNR and MSSIM metrics, and compared it with the unoptimized case and Xu’s method. Simulation results show that it can produce better recovery quality in comparison with the other two methods especially at a low SR. We also verify the extensibility of the proposed scheme and the ability to reconstruct grayscale images. We believe that the proposed scheme can promote the development and applications of GI, for example binary sampling GI.

## Supplementary Information

Supplementary Information.
