# Supplementary material for: Improving the performance of ghost imaging via measurement-driven framework
Source: Sci Rep. 2021 Mar 24;11:6776. doi: 10.1038/s41598-021-86275-2 (PMC7990946; doi:10.1038/s41598-021-86275-2)
Supplement: Supplementary file 1 — Supplementary Information. [file 41598_2021_86275_MOESM1_ESM.pdf]

Improving the performance of ghost imaging via measurement-driven framework: supplementary material

Hanqiu Kang[1], Yijun Wang[1], Ling Zhang[1,*], and Duan Huang[2]

[1]School of Automation, Central South University, Changsha 410083, China
[2]School of Computer Science and Engineering, Central South University, Changsha 410083, China
[*]lingzhang2019@csu.edu.cn

ABSTRACT

This supplement contains the alternating update process of sparse basis and sampling matrix.

Supplementary S1

Update sparse basis. For Φ_{i-1} fixed, $\rho(\Phi_{i-1},\Psi_i)$ can be rewritten as

$$\begin{aligned}\rho(\Phi_{i-1},\Psi_i) &= \|(I_N-\omega\Phi_{i-1}^{\top}\Phi_{i-1})(X-\Psi_i\widehat{Z})\|_F^2 \\ &= \|(I_N-\omega\Phi_{i-1}^{\top}\Phi_{i-1})X-(I_N-\omega\Phi_{i-1}^{\top}\Phi_{i-1})\Psi_i\widehat{Z}\|_F^2 \\ &= \|A_i-B_i\Psi_i\widehat{Z}\|_F^2\end{aligned} \tag{1}$$

where both

$$A_i=(I_N-\omega\Phi_{i-1}^{\top}\Phi_{i-1})X, \quad B_i=I_N-\omega\Phi_{i-1}^{\top}\Phi_{i-1} \tag{2}$$

are known and independent of Ψ_i. Therefore,the problem of updating sparse basis is equivalent to

$$\Psi_i=\arg\min_{\Psi_i}\ \|A_i-B_i\Psi_i\widehat{Z}\|_F^2 \tag{3}$$

which can be solved by the following derivation process.

By applying singular value decomposition (SVD) to B_i and $\widehat{Z}$, we can get

$$B_i=U_B\begin{bmatrix}\Sigma_B & 0\\ 0 & 0\end{bmatrix}V_B^{\top}, \quad \widehat{Z}=U_Z\begin{bmatrix}\Sigma_Z & 0\\ 0 & 0\end{bmatrix}V_Z^{\top} \tag{4}$$

Then, the solution to Eq.(3) is given as

$$\Psi_i=V_B\begin{bmatrix}\Sigma_B^{-1}\widehat{A}_{11}\Sigma_Z^{-1} & \widehat{\Psi}_{12}\\ \widehat{\Psi}_{21} & \widehat{\Psi}_{22}\end{bmatrix}U_Z^{\top} \tag{5}$$

where $\widehat{A}_{11}$ is corresponding to the first block of

$$\widehat{A}=U_B^{\top}A_iV_Z=\begin{bmatrix}\widehat{A}_{11} & \widehat{A}_{12}\\ \widehat{A}_{21} & \widehat{A}_{22}\end{bmatrix} \tag{6}$$

with dimension $rank(B_i)\times rank(\widehat{Z})$, and $\widehat{\Psi}_{12},\widehat{\Psi}_{21}$ and $\widehat{\Psi}_{22}$ are determined by

$$\Psi_{i-1}=\begin{bmatrix}\widehat{\Psi}_{11} & \widehat{\Psi}_{12}\\ \widehat{\Psi}_{21} & \widehat{\Psi}_{22}\end{bmatrix} \tag{7}$$

Supplementary S2

Update sampling matrix. For Ψ_i fixed, $\rho(\Phi_i, \Psi_i)$ can be rewritten as

$$\begin{aligned}\rho(\Phi_i, \Psi_i) &= \|(I_N - \omega\Phi_i^\top\Phi_i)(X - \Psi_i\widehat{Z})\|_F^2 \\ &= \|X - \Psi_i\widehat{Z} - \omega\Phi_i^\top\Phi_i(X - \Psi_i\widehat{Z})\|_F^2 \\ &= \|E_i - \omega\Phi_i^\top\Phi_iE_i\|_F^2\end{aligned} \tag{8}$$

where $E_i = X - \Psi_i\widehat{Z}$ is the sparse representation error (SRE) for the i-th iteration. Thus, the problem of updating sampling matrix is transformed into

$$\Phi_i = \arg\min_{\Phi_i} \left\|E_i - \omega\Phi_i^\top\Phi_iE_i\right\|_F^2 \quad \text{subject to} \quad \Phi_i = U\,[I_M\ 0]\,V^\top \tag{9}$$

which can be approximately solved as follows.

We perform SVD on E_i as

$$E_i = U_ES_EV_E^\top \tag{10}$$

then, substitute Eq.(10) and the constraint condition of sampling matrix in Eq.(9) into Eq.(8) and obtain the following equation:

$$\begin{aligned}\rho(\Phi_i, \Psi_i) &= \left\|U_ES_EV_E^\top - \omega V \begin{bmatrix} I_M \\ 0 \end{bmatrix} U^\top U \begin{bmatrix} I_M & 0 \end{bmatrix} V^\top U_ES_EV_E^\top\right\|_F^2 \\ &= \left\|U_ES_E - \omega V \begin{bmatrix} I_M & 0 \\ 0 & 0 \end{bmatrix} V^\top U_ES_E\right\|_F^2\end{aligned} \tag{11}$$

Due to the difficulty to solve the minimization problem in Eq.(9), an alternative is to find a feasible solution. Here, let $V = U_E$, and we can get

$$\Phi_i = U\,[I_M\ 0]\,U_E^\top \tag{12}$$

where U is an arbitrary orthonormal matrix with proper dimensions.
